# Estimation of SPIO Nanoparticles Uptakes by Macrophages Using Transmission Electron Microscopy

**DOI:** 10.3390/ijms232213801

**Published:** 2022-11-09

**Authors:** Adham Aleid, Khalid Alhussaini, Mohammed Almijalli, Ali S. Saad

**Affiliations:** Department of Biomedical Technology, King Saud University, Riyadh 11433, Saudi Arabia

**Keywords:** iron oxide nanoparticles, drug delivery, electron microscopy, segmentation, image processing

## Abstract

Due to their interesting size-dependent magnetic characteristics and relative biocompatibility, magnetic superparamagnetic iron oxide (SPIO) nanoparticles have been widely exploited as probes for cell and subcellular structure identification, as well as medication and gene delivery. A thorough understanding of the mechanics of the interaction between nanoparticles and macrophages is vital in managing dynamic processes in nanomedicine. In this study, the interaction behavior and uptake of SPIO nanoparticles by M1- and M2-type macrophages were investigated. Mice monocytes were differentiated into M1 and M2 macrophages, and the uptake of SPIO nanoparticles was studied using a TEM microscope. A high resolution image of 1 nm resolution, an image processing technique, was developed to extract the SPIO-NPs from tomographic TEM microscopic images. Lysosomes appear to be the zones of high concentrations of SPIO inside macrophages. Lysosomes were first selected in each image, and then segmentation by the Otsu thresholding method was used to extract the SPIO-NPs. The Otsu threshold method is a global thresholding technique used to automatically differentiate SPIOs from the background. The SPIO-NPs appear in red colors, and the other pixels in the image are considered background. Then, an estimation of the SPIO-NP uptakes by lysosomes is produced. Higher uptake of all-sized nanoparticles was observed in M1- and M2-type macrophages. An accurate estimation of the number of SPIO-NPs was obtained. This result will help in controlling targeted drug delivery and assessing the safety impact of the use of SPIO-NPs in nanomedicine for humans.

## 1. Introduction

Because of rapid advancements in nanotechnology and the shrinking size of features in the biological sciences, advanced characterization with high spatial resolution in two and three dimensions is required [1]. More relevant quantitative analysis is possible with electron microscopy, which uses tomography to rebuild the three-dimensional nature of an object from a tilt series of two-dimensional projections (pictures) [2]. Electron tomography has a resolution of around 1 nm and a field of view of hundreds of nanometers, making it excellent for characterizing a wide range of nanoscale objects. This is the most extensively used electron microscopy approach for acquiring three-dimensional information.

Nanoparticles are widely used in biomedical applications [3]. In some applications, to investigate certain properties or efficacy, nanoparticle uptake by cells is studied. For example, nanoparticles are mostly taken up by the reticulo-endothelial system (RES), which is made up primarily of macrophages [4] and is involved in the coordination of immune responses, pathogen clearance, and tissue homeostasis management.

Thus, NP drug delivery to solid tumors is hampered by a number of biological hurdles caused by the in vivo system’s complexity. NPs are typically given intravenously (IV) as part of modern anti-cancer therapy. This method is quick and dependable and permits full dispersion across systemic circulation. NPs encounter a variety of difficulties once they are in use. Blood proteins may make them more susceptible to phagocytosis, which allows the mononuclear phagocyte system (MPS) to identify and eliminate them from circulation [5]. The NP population that the MPS was unable to remove must now be extravagated out of circulation successfully through the endothelium lining and into the tumor microspace. The second barrier is thus effective extravasation, which is followed by the tumor interstitial as the penultimate barrier. In addition to several physiological variables, including low pH, low oxygenation, and high interstitial fluid pressure, the NP comes into contact with smooth muscle cells, extra-cellular matrix, pericytes, and cancer-associated fibroblasts. The tumor cell membrane and intracellular machinery are the ultimate barriers the NPs must pass for the successful intracellular distribution of drug cargo once they have extravagated out of systemic circulation, past the tumor microspace, etc. Therefore, it is essential to construct multifunctional NPs with layers of specialized properties that can progressively execute functions to break through these biological barriers one at a time [6]. 

Certain research is aimed at determining the unfavorable, unintended, or harmful effects of NPs once they have been administered to the human body. The study in [7] attempted to understand and exploit NPs’ interaction with the blood vessel and blood in order to identify side effects that may occur in vivo during the delivery of NPs to the area of interest. Another study [8] looked into the physicochemical properties of NPs in order to better understand how nanoparticles affect cell biology. In addition, the phenomenon of NP aggregation in cells may have an impact on drug delivery, diagnostic imaging, or hazardous potential. Ref. [9] proposed a quick screening approach for evaluating NP aggregation in the blood.

After exposure to TiO_2_ and SiO_2_ NPs, epithelial cell sheet movement slowed, and wound healing ability was considerably reduced. “Cells after exposure to the nanoparticles showed increased cell contractility with significantly impaired wound healing capability but without any apparent cytotoxicity. They showed the mechanism is through nanoparticle-mediated massive disruption of the intracellular microtubule assembly, thereby triggering a positive feedback that promoted stronger substrate adhesions thus leading to limited cell motility” [10].

Magnetic Superparamagnetic iron oxide (SPIO) nanoparticles have been widely exploited as probes for cell and subcellular structure identification, as well as medication and gene delivery [11], due to their interesting size-dependent magnetic characteristics and relative biocompatibility. They have been used in a variety of biomedical applications, including magnetic resonance imaging (MRI) and medication delivery to living cells [12,13,14]. The use of SPIO nanoparticles as contrast agents in the non-invasive imaging of macrophage activity has piqued interest in the diagnosis of disorders involving inflammation, infections, and tissue degeneration, making them potential carriers for delivering diagnostic or therapeutic contrast agents [15]. Distinct environmental variables inside the body cause macrophages to take on different activities or polarization states [16,17]. M1 macrophages, which have been stimulated in the past, have a pro-inflammatory effect. Alternatively, activated or M2 macrophages, on the other hand, exhibit immunomodulatory properties and promote wound healing and angiogenesis [18]. TEM visualization and quantification are used to filter NP aggregates, manage the drug and gene delivery process, and improve the diagnosis and treatment of many diseases using MRI imaging [19,20].

Targeting macrophages using nanoparticles has recently been used as a clinical therapeutic agent against cancer and inflammation, a novel avenue for cancer drug discovery and a potential therapeutic strategy for atherosclerosis [21,22,23,24,25,26].

In [27], the interactions of nanoparticles with macrophages and the feasibility of drug delivery for asthma were studied. They targeted gold delivery effectiveness using a mouse model of asthma triggered by ovalbumin and examined the biodistribution of nanoparticle-loaded macrophages. Macrophage mobility was higher in models of severe asthma than in models of mild asthma. In [28], they tried to understand the modes of interaction between human monocytes/macrophages and engineered nanoparticles to assess particle safety, in terms of the activation of innate/inflammatory reactions, and their possible exploitation for medical applications. In [29], they used models of breast cancer in three immune variants of mice and demonstrated that intra-tumor retention of antibody-labeled nanoparticles was determined by tumor-associated dendritic cells, neutrophils, monocytes, and macrophages and not by antibody-antigen interactions. Nanoparticle–macrophage interactions, a balance between clearance and cell-specific targeting, were studied in [30], and they found that acetylation was comparable to PEGylation in reducing RES clearance. Additionally, they found that dendrimer acetylation did not impact folic acid (FA)-mediated targeting of tumor cells, whereas PEG surface modification reduced the targeting ability of NPs. As a result, deeper knowledge of the mechanics of the interaction between nanoparticles and macrophages will help us manage dynamic processes in nanomedicine much more effectively. Therefore, an accurate estimation of the quantity of NP uptakes by macrophages using TEM imaging and image processing will provide better control of drug delivery and safety considerations for therapeutic purposes, as well as for diagnostic ones.

In this paper, an image processing method for quantifying SPIO-NP absorption by macrophages is proposed. It is based on TEM tomographic imaging for two- and three-dimensional reconstructions with high-resolution images (1 nm per pixel). To aid researchers in calculating the amount of NPs absorbed by macrophages for safety, therapy, diagnosis, and control of drug delivery considerations.

## 2. Results

**Figure 1:**
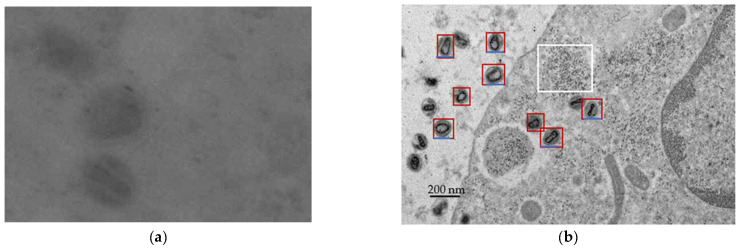
Transmission electron microscopy micrographs; (**a**) a control image without SPIO-NP. (**b**) AMNP-labeled M2 macrophages revealing the intralysosomal localization of nanoparticles or microvesicles (100 nm–1 μm), which directly bud from the plasma membrane. The red box englobes a lysosome or a microvesicle. The black scale bar on the left corresponds to 200 nm, and the blue scale bar represents 100 nm.

Figure 1a shows a sample of the control tomographic tilt series, and Figure 1b shows the macrophage with SPIO1-M1 obtained from the TEM Hitachi H7500.

**Figure 2:**
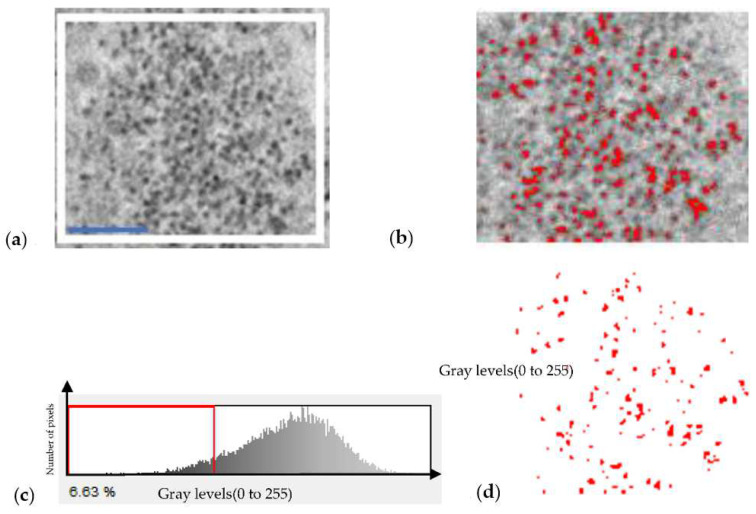
The white rectangle region from the background extracted from Figure 1. (**a**) The original image with a scale bar of 100 nm. (**b**) Image after selection of the SPIO nanoparticles in red color, (**c**) SPIOs were extracted from (**b**) using threshold method, and (**d**) is the threshold selected at the first minimum of the histogram.

The background image in Figure 2 shows the individual SPIO in the background from which the size of the SPIO is estimated. The Otsu method was utilized to determine the threshold. The SPIO-NPs are represented by the red pixels in (b), where (c) includes the red particles representing the SPIOs and (d) the threshold used to separate the NPs is shown. The quantity of red pixels in each lysosome estimates how many SPIO-NPs the macrophage has ingested if we can count the total number of lysosomes and/or microvesicles in the macrophage.

**Figure 3:**
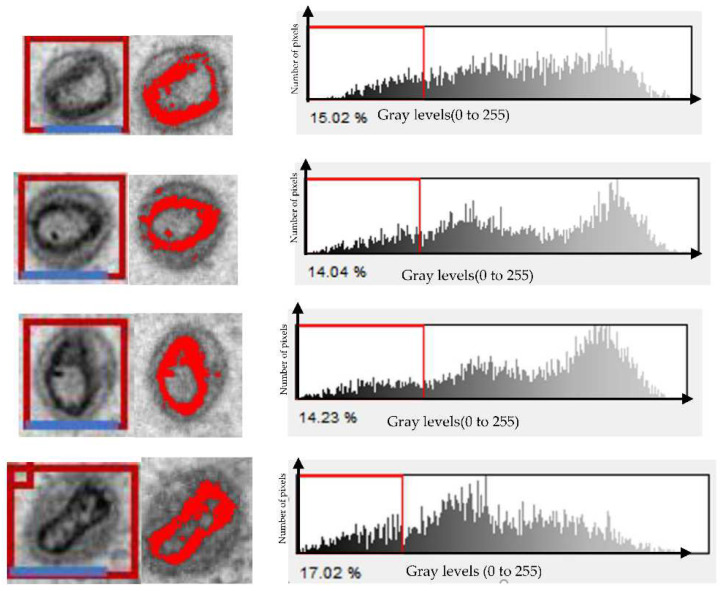
The first column represents 4 single microphages after uptake of the SPIO nanoparticles. The blue scale bar on the first column of the images represents 100 nm. The second column represents the segmented regions in red color where the SPIOs NPs inside the lysosomes or microvesicles. The third column is the histogram of the image. The red line represents the threshold chosen at the first minimum. The vertical axis represents the number of pixels for each gray level. The horizontal axis represents gray levels (0 to 255).

Figure 3 shows the lysosomes/microvesicles in the first column; they seem slightly of different sizes, and the diameters vary between 160 and 250 nm. The SPIO NPs are represented by the red dots in column 2 of Figure 3. In the picture, varying amounts of NPs are shown in red inside each lysosome. SPIOs are seen inside the lysosomes in these images. Therefore, it is simple to estimate the amount of SPIOs engulfed by each lysosome/microvesicles by counting the red dots or pixels inside it and then estimating the number of NPs inside macrophages by counting the total number of NPs for all lysosomes/microvesicles inside the macrophages. The image resolution and the diameter of each SPIO (10 to 13 nm) were considered. The results are shown in the last column of Table 1.

## 3. Discussion

The surface properties of NPs are a major factor that influences how these nanomaterials interact with biological systems. Interactions between NPs and macrophages of the reticuloendothelial system (RES) can reduce the efficacy of NP diagnostics and therapeutics. Traditionally, to limit NP clearance by the RES, the NP surface is neutralized with molecules such as poly(ethylene glycol) (PEG), which are known to resist protein adsorption and RES clearance [31].

One of the major barriers limiting nanotherapeutic delivery is the inability to attain therapeutic levels at the target tissue because of NP clearance by the RES. The RES system, which consists predominantly of macrophages, functions to sequester and clear NP after their administration [32]. In this paper, we estimate the number of NPs absorbed by lysosomes inside macrophage cells.

The tomographic TEM pictures have a resolution of around 10 (9.7) angstroms per pixel (1 nm), and the NPs employed have an average diameter of about 11.5 nm. In this work, the size of the processed image was reduced by 4 for the RAM memory issue, and for reducing the processing time, which makes the resolution decrease four times, it became 40 angstroms instead of ten, reduced. The surface covered by NPs inside this macrophage can be estimated by counting the red pixels in the lysosomes [33] inside the macrophages. A single SPIO-NP surface area is calculated using (πd^2^/4) where d = 11.5 nm is the average diameter of the SPIO-NP. The surface area of an average SPIO is about 6 nm^2^ according to the actual resolution of the TEM image used after size reduction by 4. The total number of NPs inside the lysosomes can be estimated by counting the red pixels and dividing the result by the surface area of one SPIO (see Table 1). The average number of SPIO-NPs absorbed by one lysosome is about 78. Using MRI scans, SPIO-NPs assist in locating the position of macrophages. Because of the SPIO magnetic properties, MRI images have higher intensities at the locations of clusters of SPIOs [34]. Macrophages are used as drug delivery vehicles, and SPIO-NPs can enable MRI imaging to detect macrophage intensities and locations inside the target organ. As macrophages are connected with the drug on top of it, this will allow us to estimate the quantity of drug delivered to the area of interest. By calculating the amount of drug received by the site of interest, this visualization and quantification increases the accuracy of drug delivery (inflammation or cancer). It can also aid in nanomedicine diagnosis and therapeutic procedures. Seeing the NPs inside macrophages in 2D and estimating their approximate number would undoubtedly aid in reducing the negative effects of NPs, as well as medication delivery and imaging processes for nanomedicine applications.

## 4. Materials and Methods

### 4.1. Macrophage Polarization and Labeling

As previously described [30], bone marrow (BM)-derived M1 and M2 macrophages (BMDM) were obtained.

For effective macrophage labeling, dextran-coated iron oxide nanoparticles functionalized with PEG were selected [35,36]. These nanoparticles have previously been shown to have improved macrophage labeling effectiveness while also being more biocompatible [33]. According to the supplier’s specifications, these nanoparticles have an dextran coating of 40,000 g/mol and a PEG length of 300 g/mol, resulting in a total diameter of 100 nm and an iron oxide crystallite diameter of 10–13 nm. Prior to incubation with the cells, the SPIO-NPs were exposed to UV light for 30 min. Then, cells were incubated for 30 min at 37 °C with SPIO-NPs in serum-free RPMI culture medium supplemented with 5 mM citrate sodium. Free citrate anions ensured the colloidal stability of SPIO-NPs in RPMI medium. The extracellular iron concentration was 2 mM. To allow enough time for iron oxide internalization before fixation for one hour at room temperature for TEM tomographic investigation, the incubation stage was followed by 4–5 h in SPIO-free culture media.

In serum-free RPMI medium, M1- and M2-polarized macrophages were incubated for 30 min with carboxyl- or amine-modified PEGylated SPIO nanoparticles (SPIO-PEG-COOH or SPIO-PEG-NH2, respectively) to avoid protein interactions with the uptake mechanism [37], as the colloidal stability of particles in protein-rich media can be influenced by interface characteristics [38].

Zeta potential measurements of the SPIO in cell culture media at concentrations of 2 mM were described in [33]. The electrical surface charge of the SPIO nanoparticles was determined by measuring the zeta potential using a Zetasizer Nano ZS90 (Malvern Instruments, Malvern, UK). These nanoparticles were tested at a concentration range of 0.01–1 mg/mL in ultrapure water at pH 7.4. The average of three measurements was taken, and the results were expressed as zeta potential. Zeta potential analysis has revealed that SPIO, SPIO–PEG, and SPIO–PEG–COOH have a surface charge of −1.8, −3.3, and −5.8 mV, respectively [33].

Iron-labeled macrophages were then rinsed and re-suspended in PBS. This protocol allows the production of approximately 5 million activated and labeled M1 or M2 macrophages [30].

### 4.2. TEM Tomography Imaging and Sample Preparation

A total of 10 TEM images were used in this study. M1 and M2 macrophages were grown and tagged in Permanox dishes so that the cells could be processed directly without scraping. They were fixed in 0.05 M sodium cacodylate buffer (pH 7.4) with 2.5 percent glutaraldehyde, then post-fixed with 2 percent osmium tetroxide, dehydrated in escalating degrees of ethanol, and lastly embedded in Epon resin. A Hitachi H7500 transmission electron microscope operating at 80 kV (National Center for Macromolecular Imaging, Baylor College of Medicine, TX, USA) was used to image ultrathin sections (70 nm) with a tilt series (−60° with 2° increment step size) at a resolution of 9.7 Angstrom/pixel with a time of exposure of 1 s and a defocus of 0 m. Golden particles of size 15 nm were used as fiducial seeds for alignment purposes.

### 4.3. Segmentation Method Using the Otsu Threshold

In this work, the segmentation method is used to partition image pixels into two categories: background and object [39]. A local threshold, on the other hand, is applied to a small section of the image. In addition, establishing the proper threshold value involves prior knowledge of the image’s content. The SPIO pixel threshold value can be determined using the Otsu method based on the intensity histogram as well as other statistical data. The Otsu threshold method is a global thresholding technique that is used to automatically apply a threshold to photographs. As seen in Figure 3, the method first builds a histogram for the image.

The algorithm exhaustively searches for the threshold that minimizes the intra-class variance, defined as a weighted sum of variances of the two classes.

The intensity variance is then calculated, and the threshold that minimizes the intra-class variance is used as the Otsu threshold.

Pixels with a higher intensity than the threshold value are set to 255 (white color), while pixels with the lowest intensity are set to 0 (red color). Pixels are separated into two groups as a result of Otsu’s algorithm: foreground and background.

h(i,j) = 255 (white color) if f(i,j) ≥ T; and

h(i,j) = 0 (red color) if f(i,j) < T

where f(i,j) represents the original image’s pixel intensity, T represents the final Otsu threshold value, and h(i,j) represents the final segmented image.

Matlab and ImageJ softwares (https://imagej.nih.gov/ij (accessed on 12 March 2022)) were used to accomplish the processing (Supplementary Materials—Algorithm S1, Otsu Algorithm).

## 5. Conclusions

This work has shown that macrophage SPIO interaction can be quantified using tomographic TEM high-resolution images. On average, each lysosome inside the macrophage can absorb less than hundred SPIO. It also shows that high concentrations of the SPIOs are located inside the lysosomes, which provides a good intensity to be seen in MRI images. Hence, this study could help in assessing the quantity of SPIOs inside the macrophages in a 2D image. This provides researchers with a more accurate framework for drug delivery, diagnosis imaging, safety effects of NPs, and other nano-medicine applications. Future work will provide a more accurate and full estimation of SPIO-NPs inside macrophages. The study can be accomplished if we can use the full resolution of the TEM image with a super computer having a high capacity of RAM memory.

## Figures and Tables

**Table 1:** The results of 8 images of lysosome/microvesicles where the percentage and the estimated number of SPIO-NPs is shown in the last 2 columns.

Lysosome Image Number	Total Number of Pixels	Number of Red Pixels	% of Red Pixels	Estimated Number of SPIO
1	3654	548	15%	93
2	3782	529	14%	88
3	4140	589	14.23%	98
4	2950	502	17%	84
5	3016	431	14.32%	72
6	3762	412	11%	70
7	2860	496	17.34%	86
8	2700	324	12%	40
Average	3358	479	14%	78

## Data Availability

Not applicable.

## Supplementary Materials

The following supporting information can be downloaded at: https://www.mdpi.com/article/10.3390/ijms232213801/s1. Algorithm S1—Otsu's method.
