# Supplementary material for: Estimation of SPIO Nanoparticles Uptakes by Macrophages Using Transmission Electron Microscopy
Source: Int J Mol Sci. 2022 Nov 9;23(22):13801. doi: 10.3390/ijms232213801 (PMC9692596; doi:10.3390/ijms232213801)
Supplement: Supplementary file 1 [file ijms-23-13801-s001.zip › ijms-1970255-supplementary.pdf]

Algorithm S1—Otsu's method

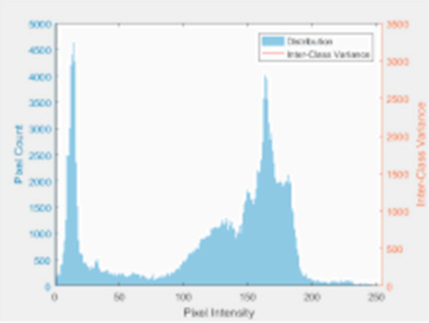

Otsu's method visualization

The algorithm exhaustively searches for the threshold that minimizes the intra-class variance, defined as a weighted sum of variances of the two classes:

$$\sigma_w^2(t) = \omega_0(t)\sigma_0^2(t) + \omega_1(t)\sigma_1^2(t)$$

Weights $\omega 0$ and $\omega 1$ are the probabilities of the two classes separated by a

threshold ,and $\sigma 0$ and $\sigma 1$ are variances of these two classes.

The class probability is computed from the L bins of the histogram:

$$\omega_0(t) = \sum_{i=0}^{t-1} p(i)$$

$$\omega_1(t) = \sum_{i=t}^{L-1} p(i)$$

For 2 classes, minimizing the intra-class variance is equivalent to maximizing inter-class variance:[2]

$$\begin{aligned}\sigma_b^2(t) &= \sigma^2 - \sigma_w^2(t) = \omega_0(t)(\mu_0 - \mu_T)^2 + \omega_1(t)(\mu_1 - \mu_T)^2 \\ &= \omega_0(t)\omega_1(t)[\mu_0(t) - \mu_1(t)]^2\end{aligned}$$

which is expressed in terms of class probabilities ω and class means μ, where the class means , μ_0,$\mu 1$, and μ_T are:

$$\mu_0(t) = \frac{\sum_{i=0}^{t-1} ip(i)}{\omega_0(t)}$$

$$\mu_1(t) = \frac{\sum_{i=t}^{L-1} ip(i)}{\omega_1(t)}$$

$$\mu_T = \sum_{i=0}^{L-1} ip(i)$$

The following relations can be easily verified:

$$\omega_0\mu_0 + \omega_1\mu_1 = \mu_T$$

$$\omega_0 + \omega_1 = 1$$

The class probabilities and class means can be computed iteratively. This idea yields an effective algorithm.

Algorithm

MATLAB implementation

`histogramCounts` is a 256-element histogram of a grayscale image different gray-levels (typical for 8-bit images). `level` is the threshold for the image (double).

```
function level = otsu(histogramCounts)
total = sum(histogramCounts); % total number of pixels in the image
%% OTSU automatic thresholding
top = 256;
sumB = 0;
wB = 0;
maximum = 0.0;
sum1 = dot(0:top-1, histogramCounts);
for ii = 1:top
    wF = total - wB;
    if wB > 0 && wF > 0
        mF = (sum1 - sumB) / wF;
        val = wB * wF * ((sumB / wB) - mF) * ((sumB / wB) - mF);
        if ( val >= maximum )
            level = ii;
            maximum = val;
        end
    end
    wB = wB + histogramCounts(ii);
    sumB = sumB + (ii-1) * histogramCounts(ii);
end
end
```
